# Mineral and Trace Element Analysis of Australian/Queensland *Apis mellifera* Honey

**DOI:** 10.3390/ijerph17176304

**Published:** 2020-08-29

**Authors:** Natasha L. Hungerford, Ujang Tinggi, Benjamin L. L. Tan, Madeleine Farrell, Mary T. Fletcher

**Affiliations:** 1 Queensland Alliance for Agriculture and Food Innovation (QAAFI), The University of Queensland, Health and Food Sciences Precinct, Coopers Plains, QLD 4108, Australia; mary.fletcher@uq.edu.au; 2 Forensic and Scientific Services, Queensland Health, Coopers Plains, QLD 4108, Australia; Ujang.Tinggi@health.qld.gov.au (U.T.); Benjamin.Tan@health.qld.gov.au (B.L.L.T.); Madeleine.Farrell2@health.qld.gov.au (M.F.)

**Keywords:** *Apis mellifera*, honey, Australia, Queensland, trace metals, minerals, heavy metals

## Abstract

Honey is an extensively utilized sweetener containing sugars and water, together with small quantities of vitamins, minerals, fatty acids, amino acids and proteins. Naturally produced by honeybees (*Apis mellifera*) from floral nectar, honey is increasingly sold as a health food product due to its nutritious features. Certain honeys are retailed as premium, trendy products. Honeybees are regarded as environmental monitors, but few reports examine the impact of environment on Australian honey trace elements and minerals. In higher density urban and industrial environments, heavy metals can be common, while minerals and trace elements can have ubiquitous presence in both agricultural and urban areas. Honey hives are traditionally placed in rural and forested areas, but increasingly the trend is to keep hives in more urban areas. This study aimed to determine the levels of 26 minerals and trace elements and assess elemental differences between honeys from various regional Queensland and Australian sources. Honey samples (n = 212) were acquired from markets, shops and supermarkets in Queensland while urban honeys were purchased online. The honey samples were classified into four groups according to their regional sources: urban, rural, peri-urban and blend honey. Elemental analyses of honey were performed using ICP-MS and ICP-OES after microwave and hot block digestion. Considerable variations of essential trace elements (Co, Cu, Cr, Fe, Mn, Mo and Zn) and mineral levels (Ca, K, Mg, Na and P) were found in honeys surveyed. There were significant differences (*p* < 0.05) between urban and rural honey samples for B, Na, P, Mn, K, Ca and Cu. Significant differences (*p* < 0.05) were also found between blend and urban honey samples for K, Cu, P, Mn, Sr, Ni, B and Na. Peri-urban versus urban honeys showed significant differences in P, K and Mn. For rural and peri-urban honeys, the only significant difference (*p* < 0.05) was for Na. Toxic heavy metals were detected at relatively low levels in honey products. The study revealed that the Queensland/Australian honey studied is a good source of K and Zn and would constitute a good nutritional source of these elements.

## 1. Introduction

Honey and other bee products are widely consumed as food with unique nutritional and medicinal properties, but contamination by anthropogenic activities may bring health hazards [1] from pesticide, antibiotic, heavy metal and microbial contamination. The exact composition of honey is complex and dependent on the geographic location, botanical source(s), season, storage and processing conditions. Quality standards (chemical and physical specifications) for honey are prescribed in the internationally recognized Codex standard [2], while the FSANZ honey standard [3] describes basic characteristics. Honey contains a wide variety of minerals and trace elements, influenced by botanical and geographical origins, and it can be a good source of minerals and trace elements needed in the human diet. Australian honey production relies greatly on the availability of mostly rural, native bush vegetation, and it is the honey from native species, including an array of eucalypts and rainforest species, that is most valued. Hives are often transferred from one locality to the next, following the progression of flowering, with drought conditions further necessitating this [4]. By comparison, Australian honey from pollination services of agricultural crops is often less valued. An increasingly prized honey is produced locally in cities and surrounds, with a recent trend being the emergence of urban honey as an artisan product. In urban environments, there is an abundant supply of nectar, pollen and water, often even in periods of drought. The presence of abundant and varied resources for bees in these environment places them in close proximity to human activities, with associated accumulation of particular trace elements and contaminants that can be a cause of concerns over the safety of honey consumption [5,6].

In general, the mineral content of honey is a positive nutritional feature, but it could be representative of environmental pollution or the geographical origin of honey [7]. Some heavy metals are essential nutrients but can be toxic in higher amounts [8]. Minerals, heavy metals and trace elements are naturally present in the environment, as well as introduced by anthropogenic activities. Elemental levels in honey reflect diverse sources of plant, bee, honey and hive exposure. Plant elemental levels reflect local soil, water and air, and when bees collect nectar/pollen these elements are transferred, contributing to the levels in honey. Passive uptake occurs during the bees’ travels and from the hive surroundings, and contamination may also be introduced at different levels during honey storage and processing. Highly poisonous heavy metals such as Pb, Hg and Cd can cause severe health effects from acute or chronic exposures, and the presence of these toxic metals in honey is influenced by environmental pollution [9].

Forager bees can fly up to 5 (or 10) km over large areas of habitat, which will increase their exposure to environmental chemicals including trace elements and toxic metals. Thus, in more highly polluted areas, honey will reflect pollutants found in or on forage plants, in the soil and water and in the air [10,11]. Numerous studies have found As, Pb, Cd, Cr, Fe, Cu, Ni, Zn and Hg in honeybees, honey and their other products, all used as environmental monitors [12,13,14,15,16,17,18,19]. Observed differences in rank order in heavy metal accumulation along the urban–suburban gradient in honeybees [20] can have implications for the levels in honey. In one study comparing honey samples from contaminated and uncontaminated areas, mercury levels ranged 0.050–0.212 and 0.001–0.003 mg/kg, respectively [21]. Use of honey as a biomonitor in Vancouver found elevated trace elements (Pb, Fe, Ti, Zn, Cd, Al, Cr, Cu and Sb) in urban downtown areas and Pb isotope composition reflected vicinity to shipping ports/heavy traffic [15]. Similarly, Perna reported that higher Pb, Cd and Cr levels in honeys from southern Italy were linked to activities related to petroleum extraction [18]. Mineral and trace element content in honey was compared in honeys of different botanical origin, but high levels of Co, Pb and Ni were attributed to an area’s geochemistry or a polluted environment [19]. Toxic heavy metals bioaccumulate in those who consume them, and, although there are no specific MRLs for honey, values have been set by the Australian Food Code for certain foods [22]. Monitoring of honey for mineral content, including toxic heavy metals, is vital for human health and honey quality control.

Trace element analysis finds applications in food authentication, particularly in combination with stable isotope analysis [23] and in biomonitoring [24]. Complex matrices routinely analyzed for mineral and trace elements include food, environmental samples, biological samples, geological and industrial samples and require suitable digestion techniques for complete decomposition of samples for efficient elemental analysis [25]. For food matrices, wet digestion including microwave-assisted digestion and ashing techniques are widely used for dissolution of samples prior to analysis using instrumental methods such as inductively coupled plasma atomic emission spectrometry (ICP-AES), inductively coupled plasma mass spectrometry (ICP-MS), flame atomic absorption spectrometry (FAAS), electrothermal atomic absorption spectrometry (ETAAS), X-ray fluorescence spectrometry (XRF) and neutron activation analysis (NAA) [26].

In the present study, honey purchased in Queensland and honey produced in urban Australian environments were subjected to elemental analyses, to assess if land-use origins of honey influence the mineral levels. Honey purity and origin exerts a significant influence on the commercial value of the product and is of utmost importance to consumers of Australian honey. There are few studies of the composition of Australian honey [13,27,28,29]. The levels identified herein constitute a first Australian study to identify mineral levels in honey and compare honey from urban versus peri-urban versus rural settings, with blended honeys. Thus, the main aims of the study were: (1) to conduct comprehensive elemental analysis of Australian honeys; (2) to assess for any elemental differences between regional sources; and (3) to provide further information on levels of essential elements for Australian food composition databases. 

## 2. Materials and Methods

### 2.1. Sample Collection 

Honey samples (n = 212) were purchased between September 2016 and March 2018 from Queensland supermarkets, fruit shops, local markets and producers or via online suppliers. Honeys were classified into four groups as urban, rural, peri-urban or blend based on the available information for their regional origins. Urban honeys are those produced within densely populated metropolitan areas (towns, cities and suburbs); peri-urban honeys are from less densely populated areas that surround metropolitan areas (neither urban nor rural in the conventional sense); rural or countryside honeys are generated in geographical areas outside towns and cities and can encompass agricultural and forestry areas; and blend honeys were those of no specified regional origin, and were presumed to represent composite, blended honeys.

### 2.2. Elemental Analysis

Trace Elements (Ag, Al, As, B, Ba, Cd, Co, Cr, Cu, Fe, Hg, Mn, Mo, Ni, Pb, Sb, Se, Sn, Sr, V and Zn) in honeys were analyzed by ICP-MS (Agilent 8800 Triple Quad, Japan) after microwave digestion (CEM MarsXpress, USA). Minerals (Ca, K, Mg, Na and P) were analyzed by ICP-OES (Agilent 700 Series, Japan) after hot block digestion (A.I. Scientific, Australia). All elemental analyses were carried out at the Queensland Health Forensic and Scientific Services Laboratory, and the methods of analysis have been previously described [23,30]. Briefly, for microwave digestion, approximately 1.0 g of sample was taken and accurately weighed into digestion tubes and 4 mL of high purity nitric acid (69% Seastar Chemicals, Canada) were added. For hot block digestion, 5 mL of nitric acid were initially added, followed by another 5 mL aliquot of nitric acid until completion when about 1 mL digested solution remained. The digested samples were diluted to 40 mL with high purity water (Aqua Cure, England) for analysis. The certified reference material and in-house reference materials (as described in the Supplementary Materials) used for quality control were treated similarly to the actual samples throughout the analysis. The recoveries of SRMs are shown in Table S1. The values of limit of reporting (LOR) were determined by analyzing a series of blank measurements (n = 20), and final LOR for each metal was calculated as 10 times the standard deviation of the blanks, based on 1.0 g of sample and diluted to final volume of 40 mL. The LOR values are shown in Table 1.

### 2.3. Statistical Analysis

Descriptive statistics (means, standard deviations and medians) and boxplot presentations with Tukey whiskers and one-way non-parametric Kruskal–Wallis ANOVA were analyzed using GraphPad Prism 8.3.1 (GraphPad Software, San Diego, CA, USA). Dunn’s post hoc multiple comparisons test compared the mean rank of each group with the mean rank of every other group, correcting for multiple comparisons using statistical hypothesis testing, giving *p* values as multiplicity adjusted values. Any difference between regions at *p* < 0.05 was considered to be statistically significant. For statistical purposes, the concentrations of trace elements with levels < LOR (mg/kg) were taken as LOR/2 mg/kg. Spearman correlations (*r_s_*) between mineral/trace element levels were analyzed and *p* values calculated at the 95% confidence interval for those elements detected in more than nine honeys at levels above LOR.

## 3. Results 

### 3.1. Mineral and Trace Elements Found in Australian Honeys 

The levels of minerals, essential trace elements and toxic heavy metals were found to vary widely and the results are summarized in Table 1. The most abundant minerals decreased in the order (mean, mg/kg): K (965) > Na (99.7) > Ca (85.2) > P (51.5) > Mg (28.7) > Zn (6.0). The ranges for the major elements were K (202–4600 mg/kg), Na (3.7–383 mg/kg), P (12.0–920 mg/kg), Mg (6.0–190 mg/kg) and Ca (21.0–270 mg/kg). Overall, significant positive Spearman correlation coefficients (*r_s_*) were observed for B/P (*r_s_* 0.665), Ba/Mn (*r_s_* 0.745), Ba/Sr (*r_s_* 0.652), Ca/K (*r_s_* 0.735), Ca/Mg (*r_s_* 0.787), Ca/Sr (*r_s_* 0.657), K/Mg (*r_s_* 0.762) and Pb/Zn(0.622) (all with *p* < 0.0001). Significant negative correlations were observed with B/Mn (*r_s_* −0.379). The Spearman correlation coefficients are shown in Table S2, together with significant *p* values. 

### 3.2. Comparison of Mineral and Trace Elements in Honeys of Urban, Peri-Urban and Rural Origins

Table 2 summarizes the results for the minerals, trace elements and heavy metals in honey samples classified as blends, or rural, peri-urban or urban in origin. The levels of trace elements are generally low, except for Zn, Fe and Mn, for which higher levels are seen in honeys from rural regions [Zn (10.90 ± 24.7 mg/kg), Fe (4.01 ± 6.98 mg/kg) and Mn (4.48 ± 5.03 mg/kg)] as compared to urban honeys (Zn (4.90 ± 11.17 mg/kg), Fe (1.576 ± 1.33 mg/kg) and Mn (2.385 ± 2.28 mg/kg)), although the difference was only statistically different for Mn (*p* = 0.0003).

**Figure 1:**
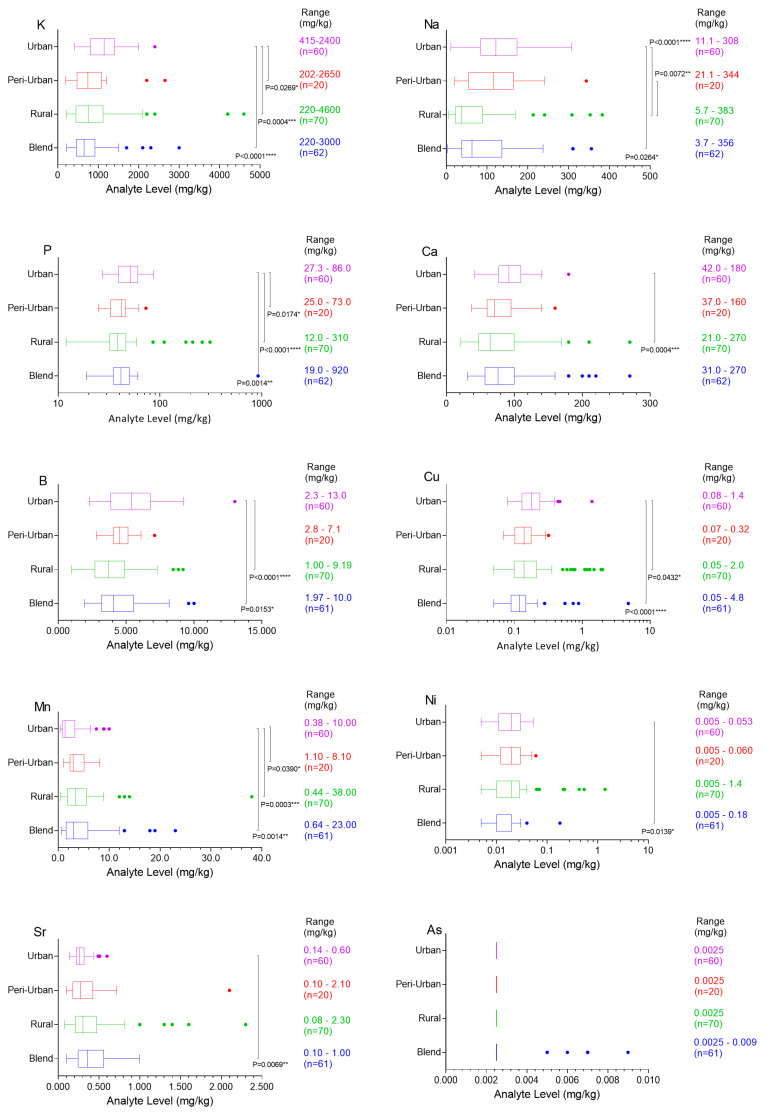
Boxplots with Tukey whiskers showing concentrations (mg/kg) for K, Na, P, Ca, B, Cu, Mn, Ni, Sr and As for honey samples categorized as urban, peri-urban, rural or blend. Significant differences were determined using one-way non-parametric Kruskal–Wallis ANOVA with Dunn’s multiple comparisons test, giving adjusted P values, **** *p* < 0.0001, *** *p* = 0.0001–0.001, ** *p* = 0.001–0.01, * *p* = 0.01–0.05, ns *p* ≥ 0.05.

The results of statistical analysis of one-way Kruskal–Wallis ANOVA with Dunn’s multiple comparisons test to assess for significant differences between honey origins are shown in Figure 1. There were significant differences between urban and rural honey samples for B (*p* < 0.0001), Na (*p* < 0.0001), P (*p* < 0.0001), Mn (*p* = 0.0003), K (*p* = 0.0004), Ca (*p* = 0.0004) and Cu (*p* = 0.043). Blend versus urban honeys showed significant differences for K (*p* < 0.0001), Cu (*p* < 0.0001), P (*p* = 0.0014), Mn (*p* = 0.0014), Sr (*p* = 0.0069), Ni (*p* = 0.0139), B (*p* = 0.0153) and Na (*p* = 0.0264). Peri-urban versus urban honeys showed significant differences in P (*p* = 0.0174), K (*p* = 0.0269) and Mn (*p* = 0.0390). Peri-urban versus rural honeys showed significant differences in Na (*p* = 0.0072).

## 4. Discussions

### 4.1. Australian Honeys Compared to Global Studies

There has been a significant interest in many countries in the elemental levels of honey products, not only in terms of nutritional importance but also for monitoring of sources of metal contaminants. In this study, wide variations of mineral levels (K, Na, P, Mg and Ca) were found in the Australian honey products. The mineral K accounted for 42–92% of the total mineral content in each honey, with a mean concentration of 965 ± 651 mg/kg, followed by Na (100 ± 83 mg/kg), Ca (85 ± 40 mg/kg), P (52 ± 67 mg/kg) and Mg (29 ± 20 mg/kg). The wide variations of 26 elements, particularly for minerals such as K, Na, P, Mg and Ca in honeys, have also been reported in many countries from diverse regions in the world (Table S3). Previous studies have also shown that K, P and Ca were the most abundant minerals found in honey [31,32]. K was observed to be the most abundant in honey from various countries including Malaysia, New Zealand, Mexico, Spain, Portugal, Italy, Poland and Slovenia [29,31,32,33,34,35,36,37,38,39,40,41,42], with mean K levels ranging from 325.54 ± 108.55 mg/kg in Croatia to 1450 ± 1100 mg/kg in Italy (Table S3). There has been little information on mineral levels (including K) in Australian honey; however, a recent study of Australian honey by Zhou and colleagues reported high mean K levels of 8370 ± 3560 mg/kg (Table S3) [29], almost 10-fold higher as compared to our results for Australian honey. These authors also reported similarly magnified K levels in honeys from other global locations when compared to those reported by other studies internationally [29,31,42,43,44,45,46].

Wide variations of essential trace element levels (Fe, Zn, Cu, Mo, Co, Mn and Cr) were also found in our Australian honeys, and the levels are comparable to values reported in other countries (Table S3). The study also found relatively higher levels of Zn (6.0 ± 16.6 mg/kg, range: 0.16–120 mg/kg) and Fe (3.1 ± 8.0 mg/kg, range: 0.20–99 mg/kg) in honey compared to other trace elements, with the largest variation observed in rural honeys. Both Zn and Fe are essential elements for plant growth and reproduction and are commonly applied in fertilizers to address Australian soil deficiencies and increase crop yield, and the variability of honey levels particularly in rural setting may reflect this anthropogenic activity. For other trace elements, the study found a relatively high level of Cr (0.06 mg/kg) in propolis honey H-PA#121, however the mean Cr level was low at 0.007 ± 0.007 mg/kg. The elevated levels of toxic metal contaminants in honey products, such as Pb, are a major concern, and in this the study relatively high levels of Pb were found from two rural honeys, one from South Australian riverlands (H-PA#86) and the other from Riverina region in New South Wales (H-PA#31) with levels of 0.69 and 0.63 mg/kg respectively. The Pb levels in these honeys were somewhat anomalous among tested Australian honeys, and the overall mean Pb level was low at 0.03 ± 0.07 mg/kg. Both honeys with high Pb levels also had high Zn levels 120 and 86 mg/kg indicative of a common source of contamination, consistent with the Spearman correlation between Pb and Zn (Table S2). South Australia is home to one of the world’s largest primary lead smelters and air-borne lead-contaminated dust is an ongoing environmental issue [47]. Recent studies have shown similar Pb contamination of honey associated with Pb levels up to 0.295 mg/kg found in honey from the mining city of Broken Hill [13] in the neighboring state of New South Wales. Pb isotope compositions were similar to local dust and ore. A study of Australian stingless bee honey found associated Pb and Zn levels, which also correlated with urban co-located soil [48]. However, the origin of the current honeys in question is distant from both these locations, and galvanized storage or processing equipment is another source of such contamination that should be considered [35,49,50,51]. 

In addition to Pb/Zn, strong Spearman correlations were seen between other elements and reflect the “natural” co-occurrence of these elements/minerals. Na, K, Mg, Ca, Sr have known co-occurrence in seawater and rainwater (particularly that deposited near the sea) [52], and thus it is not surprising to see correlations of Ca/K, Ca/Mg, Ca/Sr and K/Mg. The co-occurrence of other elemental pairs such as B/Sr/Mn in drinking water has also been recorded. Ba and Sr are alkaline earth metals and occur naturally in soil, rock and natural groundwater [53]. B is frequently deficient [54] in Australian soils, as is P, and the use of fertilizers containing both elements both in rural settings and in urban gardens may be reflected in B/P correlations (Table S2).

The mean contents of Zn (6.0 ± 16.7 mg/kg), Cd (0.003 ± 0.004 mg/kg) and Pb (0.029 ± 0.074 mg/kg) found in this study are comparable to the Polish honey values of 7.76, 0.015 and 0.048 mg/kg respectively [55]. In Croatian honey, the highest level of Pb was 2.159 mg/kg [56]; in Italian honey, the highest level of Pb was 1.390 mg/kg [18]; one Thai honey contained Pb (1.07 mg/kg) [19]; and an Iranian honey had 1.627 mg/kg of Pb [49]. High Pb levels have also been reported for Israeli honeys, which ranged from 0.15 to 8.22 mg/kg ([45], Table S3), with the highest level 12 times the highest level found in our study. The high Pb levels in honey in certain studies could be attributed to environmental pollutants including emissions from industrial sources [57]. Even though the provisional tolerable weekly intake (PTWI) for Pb was no longer considered health protective and withdrawn by WHO, there is still a need to monitor sources of Pb contaminants in foods including honey [58]. It has been estimated that the contribution of honey to Pb daily intake is about 2.92% based on the Spanish tolerable daily intakes (TDI) of 0.5 µg/kg bw/day at Pb level less than 0.1 mg/kg in honey [59]. In this study, the average Pb concentration of Australian honeys is considered low and non-threatening to human health, especially as honey is consumed in small quantities. However, at the highest Pb concentration (0.69 mg/kg) observed in honey in this study, consumption could contribute significantly to Pb daily intake. If 10 g of honey were consumed, it would exceed the US FDA provisional tolerable daily intake (PTDI) of 6 µg Pb per day for young children (0–6 years) but lower than the US FDA PTDI of 15 µg/day for older children (7+ years) [60]. The Pb daily intakes are generally low for children in many countries and in Australia it has been reported to be 0.90–11.7 µg/day [30]. In general, the levels of trace elements are low in honeys, however, high levels of Cr have been reported in Iranian honey (0.172–1.220 mg/kg) and Turkish honey (0.900 ± 0.184 mg/kg) [49]. With the exception of a recent study of Australian honeys by Zhou and colleagues [29], there is a lack of previous data on the mineral levels and trace elements composition of Australian/Queensland honeys, with a global review reporting only New Zealand honey to represent the Australian region [61]. A recent review of nine toxic element contents in honeys has shown wide variations from countries around the world [57]. Notably this global review did include a single Australian entry, which related to a single honey sample [62], and it is this paucity of published Australian data which underlies the need for the current survey.

### 4.2. Comparison of Honeys of Urban, Peri-Urban and Rural Origins

Honey samples classified as urban, peri-urban or rural in origin or as blends were compared to assess concerns related to honey produced in increasingly urban environments versus areas impacted by agricultural practice. The statistical analysis for elemental levels and origins showed significant differences between urban and rural honey samples for P, B, Na, Mn, K and Cu (Figure 1). Statistically significant differences were also shown for blend versus urban honey for K, Cu, P, Mn, B, Sr, Ni and Na. Peri-urban versus urban honeys showed significant differences in P, K and Mn. Peri-urban versus rural honeys showed significant differences in Na. Previously, Zhou [29] compared Australian mainland (n = 24) versus Tasmanian honeys (n = 7) and found significant differences in Ca (*p* < 0.001), Mg (*p* = 0.049) and Sr (*p* = 0.014). In the current study, urban honey tended to have higher K, Na, P, Ca, B and Cu compared to the other categories, particularly rural and blended honeys, suggesting that blended honey is mostly from rural/peri-urban areas. Mineral levels (K, Na, P and Ca) as well as trace elements (B and Cu) in the urban samples were clearly affected by the anthropogenic activities, with Mn and Sr on average lower in urban samples. Elevated Mn levels observed in honey from delta areas of Vancouver [15] were suggested to arise from pesticide use, but were also speculated to reflect ground or surface waters. In our Australian study, Mn as well as Sr levels were significantly lower in urban honeys compared to other honey types, which may also be consistent with pesticide use outside urban areas.

In the current study, statistical analysis was not carried out between honey origins for As, as there were insufficient data and only five honeys, classified as blends, had As levels above the LOR, with all other honeys being below the LOR. The As levels (0.003 ± 0.001 mg/kg) found in these blend honeys were considered low, and lower than reported levels (0.00054–0.69 mg/kg) in other countries (Table S3) [63]. There is no maximum permitted level of total As in the Australian Food Standards Code for honey, except for cereal products set at 1 mg/kg [3]. 

Several environmental factors could significantly contribute to variation and elevated levels of elements in honeys around the world [57]. In an earlier study, Bognadov and colleagues [64] looked at urban/non-urban, geographical and botanical influences on mineral content in Swiss honey and reported elevated values of trace elements in honey from industrial/polluted areas. Samples of 95 honeys, were collected from cities, villages, rural and mountainous areas in Switzerland. The highest average Cr levels were found in the city honey samples (0.010 mg/kg). In this Swiss analysis, levels of Pb and Cd did not depend on geographical origin of the honey and values of Pb in city honeys analyzed were relatively low. The highest levels of Pb observed were seen in village and country areas and at 0.329 mg/kg, were much higher than the acceptable limit for honey (0.10 mg/kg) as described by the European Commission [65]. Fe values were higher in city and village areas compared to rural and mountain areas. 

A study in France in 2012 [66] found honey from apiaries in hedgerow (bordering road or field) environments to be more contaminated than urban, cultivated or island settings, with the maximum Pb level observed in honey 0.378 mg/kg (mean of 0.047 mg/kg). Higher Pb levels were observed in French dry seasons. Bilandzic [67] in Croatian honey determined concentrations of As, Cd, Cu, Hg and Pb which revealed that Pb concentrations were highest in honey from hives in populated, urban and industrialized zones near highways and railways. Similarly, Tuzen [68] in Turkish honey samples observed higher levels of Cu, Mn, Zn, Ni, Se and Fe for apiaries within reach of an industrial area. In contrast, honeys remote from industry showed lower contents of Cu, Mn, Zn, Fe and Pb than did the other honeys (Pb levels of 0.0084–0.1058 mg/kg were observed). In bees themselves, heavy metal accumulation has also been shown to be higher in urban/industrial areas compared to natural reserves [16]. Thus, numerous studies have identified accompanying higher levels of trace metals in honeys from urban relative to rural settings [15,18,69].

Such obvious trends were not found in the Australian honey samples analyzed herein and may reflect the Australian preference for location of urban honey hives in backyards and on the rooftop of high-rise buildings rather than in industrial areas [70]. On average, Cu was the only heavy metal higher in urban Australian honey samples than other samples. Urban Cu sources include building materials, brake and tire wear and deposition from the atmosphere, but are usually accompanied by elevated Zn, Cd, Fe, Mn and Pb concentrations [71]. The current study found no statistical difference in levels of Zn, Fe, Pb, Cd or Cr levels based on sample origin. This may reflect the more residential nature of urban honey surrounds in Australia rather than locations near shipping ports, oil refineries or industrial precincts.

## 5. Conclusions

This study showed wide variations of elemental levels in Australian honey products, and in particular the high levels of K and Zn, which could contribute a significant dietary source for these elements. Relatively low levels of trace elements including toxic heavy metals were found in the study, and the values are comparable to other countries. The study also found significant differences between urban and rural honeys for some elements (K, Na, P, Ca, B, Cu and Mn), of which, only Mn was lower in urban honeys. Significant differences between urban and blended honeys were found for K, Na, P, B, Cu, Mn, Ni and Sr, with Mn and Sr concentrations lower in the urban honeys.

The study has provided comprehensive data on elemental levels of Australian honeys, which could be useful for assessing the quality of honey for its nutritional importance and ensures consumers’ confidence in purchasing and consuming the products. Because of the health benefits, there has been an increased interest in the consumption of honey products, and this raises concern about levels of environmental contaminants if these honeys are produced by small producers in urban areas, in the vicinity of industrial activities. It is important that elemental levels, including toxic heavy metals, are regularly monitored to ensure nutritional quality and safeguard against contaminants. The study emphasized the importance of honey elemental analysis for assessing regional variations. However, there is also a need to identify the specific source of toxic metals such as Pb. A future study should be considered and include Pb isotope composition of honey compared to local geology, for identifying the source of contamination and to rule out galvanized storage or processing equipment as an extraneous source of this contamination. 

## Figures and Tables

**Table 1:** Data summarizing minerals, trace elements and heavy metals in honey.

Element	Mean (mg/kg) ^a^	SD (mg/kg)	Range (mg/kg) ^b^	Median (mg/kg)	LOR (mg/kg)
Ag	0.005	0	0.005–0.005	0.005	<0.01
Al	1.2	1.5	0.05–14.0	0.7	<0.1
As	0.0026	0.001	0.0025–0.009	0.003	<0.005
B	4.7	2.2	1.0–19.0	4.3	<0.04
Ba	0.3	0.2	0.04–1.7	0.2	<0.01
Ca	85.2	39.9	21–270	78.5	<1.0
Cd	0.0031	0.004	0.0025–0.053	0.003	<0.005
Co	0.0167	0.044	0.005–0.48	0.005	<0.01
Cr	0.0077	0.007	0.005–0.06	0.005	<0.01
Cu	0.2	0.4	0.05–4.8	0.1	<0.05
Fe	3.1	8	0.2–99.0	1.2	<0.1
Hg	0.0025	0	0.0025–0.0079	0.003	<0.005
K	965	651	202–4600	800	<2.0
Mg	28.7	19.6	6.0–190	23.2	<1.0
Mn	3.8	4.1	0.38–38.0	2.7	<0.01
Mo	0.0115	0.029	0.005–0.29	0.005	<0.01
Na	99.7	82.5	3.7–382.7	79.2	<2.0
Ni	0.0331	0.107	0.005–1.4	0.02	<0.01
P	51.5	67.1	12–920	42	<5.0
Pb	0.0286	0.074	0.0025–0.69	0.009	<0.005
Sb	0.0051	0.001	0.005–0.01	0.005	<0.01
Se	0.0052	0.002	0.005–0.03	0.005	<0.01
Sn	0.0331	0.049	0.025–0.48	0.025	<0.05
Sr	0.4	0.3	0.08–2.3	0.3	<0.01
V	0.0052	0.001	0.005–0.02	0.005	<0.01
Zn	6	16.6	0.16–120	0.7	<0.05

^a^ Means are lower than the LOR as for statistical purposes the concentrations of trace elements with levels < LOR (mg/kg) were taken as LOR/2 mg/kg. ^b^ The lower range value is taken as LOR/2 for some elements.

**Table 2:** Minerals, trace elements and heavy metals in honey, classified as blended, rural, peri-urban or urban in origin.

Element	Blend (n = 62/61) ^a^		Rural (n = 70)		Peri-Urban (n = 20)		Urban (n = 60)	
	Mean ^b^ (mg/kg)	SD (mg/kg)	Mean ^b^ (mg/kg)	SD (mg/kg)	Mean ^b^ (mg/kg)	SD (mg/kg)	Mean ^b^ (mg/kg)	SD (mg/kg)
Ag	<LOR		<LOR		<LOR		<LOR	
Al	1.211	1.4	1.180	1.2	1.564	3.2	0.8953	0.9
As	0.0030	0.001	<LOR		<LOR		<LOR	
B	4.474	1.8	4.019	1.8	4.619	1.0	5.516	2.0
Ba	0.3118	0.24	0.3299	0.27	0.3340	0.32	0.2630	0.16
Ca	87.42	48.8	77.37	44.4	79.85	31.8	93.22	24.4
Cd	0.0027	0.001	0.0032	0.006	0.0039	0.006	0.0029	0.004
Co	0.0191	0.05	0.0231	0.06	0.0102	0.02	0.0093	0.01
Cr	0.0071	0.006	0.00700	0.005	0.0105	0.013	0.0076	0.006
Cu	0.2275	0.612	0.3104	0.429	0.1522	0.065	0.2198	0.179
Fe	3.700	12.94	4.012	6.98	2.024	2.75	1.576	1.33
Hg	<LOR		<LOR		<LOR		0.0026	0.0007
K	785.7	513	990.3	877	883.8	603	1155	408
Mg	29.10	25.0	29.29	22.0	27.49	13.5	27.80	10.4
Mn	4.592	4.64	4.480	5.03	3.560	1.98	2.385	2.28
Mo	0.0088	0.014	0.018	0.048	0.008	0.009	0.008	0.098
Na	96.26	82.40	68.91	77.4	119.9	80.4	132.9	77.8
Ni	0.018	0.022	0.058	0.184	0.022	0.014	0.022	0.011
P	55.28	112.1	50.41	50.53	41.17	12.0	51.82	13.2
Pb	0.012	0.017	0.045	0.118	0.029	0.046	0.025	0.044
Sb	<LOR		0.0051	0.00084	<LOR		0.0052	0.0009
Se	0.0054	0.0032	0.0051	0.00059	<LOR		0.0053	0.0011
Sn	<LOR		0.039	0.069	<LOR		0.028	0.013
Sr	0.421	0.219	0.420	0.376	0.406	0.432	0.285	0.094
V	0.0051	0.0006	<LOR		0.0058	0.0034	0.0052	0.0009
Zn	1.93	4.98	10.90	24.66	4.92	15.89	4.90	11.17

^a^ For one honey classified as a blend, only results for minerals were available. ^b^ Means are lower than the LOR as for statistical purposes the concentrations of trace elements with levels <LOR (mg/kg) were taken as LOR/2 mg/kg.

## Supplementary Materials

The following are available online at https://www.mdpi.com/1660-4601/17/17/6304/s1, Table S1: Recoveries of elements from standard reference materials, *Part A*. Elemental analysis by ICP-MS, *Part B*. Elemental analysis by ICP-OES, Table S2: Spearman correlation coefficients (*r*_s_) between the concentrations of elements in honey samples, Table S3: Concentration of 26 elements in honey, by country.
