# Supplementary material for: Mineral and Trace Element Analysis of Australian/Queensland Apis mellifera Honey
Source: Int J Environ Res Public Health. 2020 Aug 29;17(17):6304. doi: 10.3390/ijerph17176304 (PMC7503739; doi:10.3390/ijerph17176304)
Supplement: Supplementary file 1 [file ijerph-17-06304-s001.pdf]

International Journal of
Environmental Research and Public Health

MDPI

Supplementary material

Mineral and Trace Element Analysis of Australian/Queensland *Apis mellifera* Honey

Natasha L. Hungerford [1,*], Ujang Tinggi [2], Benjamin L. L. Tan [2], Madeleine Farrell [2] and Mary T. Fletcher[1]

[1] Queensland Alliance for Agriculture and Food Innovation (QAAFI), The University of Queensland, Health and Food Sciences Precinct, Coopers Plains, Qld, 4108, Australia; n.hungerford@uq.edu.au (N.L.H); mary.fletcher@uq.edu.au (M.T.F.);

[2] Forensic and Scientific Services, Queensland Health, Coopers Plains, Qld, 4108, Australia; Ujang.Tinggi@health.qld.gov.au (U.T.); Benjamin.Tan@health.qld.gov.au (B.L.L.T.); Madeleine.Farrell2@health.qld.gov.au (M.F.);

* Correspondence: n.hungerford@uq.edu.au

Contents

Table S1. Recoveries of elements from standard reference materials.

- *Part A.* Elemental analysis by ICP-MS
  1. Certified Reference Material (ERM - CE278k Mussel Tissue)
  2. Reference Material (FQC 305 Fish Food, In-house reference material)
- *Part B.* Elemental analysis by ICP-OES
  1. Reference Material (CRM IRMM BCR - 679 White Cabbage)
  2. Reference Material (Biota, in-house QC 301)

Table S2. Spearman correlation coefficients (r_s) between the concentrations of elements in honey samples.

Table S3. Concentration of 26 elements in honey, by country.

References

International Journal of
Environmental Research and Public Health

MDPI

Supplementary Table S1 – Recoveries of elements from standard reference materials

- *Part A*. Elemental analysis by ICP-MS

1. Certified Reference Material (ERM - CE278k Mussel Tissue)

**Element**	**This Study**			
	Mean± s.d. (mg/kg, n = 6)	Certified mean value (mg/kg)	Uncertainty (U mg/kg)	Recovery[a] (%)
Ag	0.04 ± 0.002	0.044[b]	-	91.2
Al	44.00 ± 8.56	-	-	-
As	6.46 ± 0.34	6.7	0.4	96.5
Ba	0.27 ± 0.03	-	-	-
Cd	0.31 ± 0.02	0.336	-	93.6
Co	0.20 ± 0.02	0.21	-	94.4
Cr	0.55 ± 0.02	0.73	0.22	75.9
Cu	5.75 ± 0.24	5.98	0.27	96.1
Fe	156 ± 14	161	8	97.1
Hg	0.06 ± 0.01	0.071	0.007	90.5
Mn	4.50 ± 0.29	4.88	0.24	92.2
Mo	0.59 ± 0.28	-	-	-
Ni	0.64 ± 0.05	0.68	0.15	93.4
Pb	1.97 ± 0.10	2.18	0.18	90.5
Sb	0.005 ± 0.001	-	-	-
Se	1.65 ± 0.14	1.62	0.12	102.1
Sn	0.02 ± 0.01	-	-	-
Sr	17.27 ± 2.28	19	1.2	90.9
V	0.52 ± 0.05	-	-	-
Zn	70.18 ± 4.67	71	4	98.8

International Journal of
Environmental Research and Public Health

MDPI

Supplementary Table S1 – Recoveries of elements from standard reference materials (Cont'd)

- *Part A*. Elemental analysis by ICP-MS (Cont'd)

  2. Reference Material (FQC 305 Fish Food, In-house reference material)

	**This Study**			
Element	Mean ± s.d. (mg/kg, n = 5)	Ref value (Mean mg/kg)	Uncertainty (U mg/kg)	Recovery[a] (%)
Ag	< 0.01	-	-	-
Al	224 ± 20.7	180	52	124.4
As	0.59 ± 0.02	0.62	0.06	95.8
B	3.94 ± 0.32	3.51	0.24	112.3
Cd	0.27 ± 0.01	0.30	0.02	88.5
Co	0.17 ± 0.06	0.15	0.01	109.3
Cr	1.91 ± 0.23	2.12	0.46	90.1
Cu	14.5 ± 0.52	15.8	1.00	92.1
Fe	290 ± 28	324	30	89.5
Mn	18.1 ± 0.94	19.10	0.70	94.7
Mo	0.80 ± 0.05	0.88	0.03	90.5
Ni	0.48 ± 0.03	-	-	-
Se	0.41 ± 0.02	0.43	0.06	95.4
Sn	0.02 ± 0.01	-	-	-
Sr	125 ± 5.2	-	-	-
V	0.95 ± 0.06	-	-	-
Zn	33.1 ± 1.0	35.90	2.30	92.2

International Journal of
Environmental Research and Public Health
MDPI

Supplementary Table S1 – Recoveries of elements from standard reference materials (Cont'd)

- *Part B*. Elemental analysis by ICP-OES

1. Reference Material (CRM IRMM BCR - 679 White Cabbage)

**Element**	**This study**	**BCR-679**		
	Mean ± s.d. (mg/kg, n = 6)	Certified value (mean mg/kg)	Uncertainty (U mg/kg)	Recovery[a] (%)
Ca	7891 ± 177	7768	655	101.6
K	30592 ± 2581	-	-	-
Mg	1234 ± 22	1362	127	90.6
Na	1367 ± 21	-	-	-
P	3089 ± 16	3307	240	93.4

2. Reference Material (Biota, in-house QC 301)

**Element**	**Mean ± s.d. (mg/kg, n = 6)**	**Ref value**	**Uncertainty (U mg/kg)**	**Recovery[a] (%)**
Ca	4436 ± 38	4630	190	95.8
K	2311 ± 15	2440	120	94.7
Mg	2416 ± 36	2590	100	93.3
Na	10861 ± 186	11800	400	92.0
P	5714 ± 74	6170	200	92.6

[a] Recovery (%) was calculated as: (determined mean/reference mean)*100
[b] Reference value for Ag.

Supplementary Table S2. Spearman correlation coefficients (r_s) between the concentrations of elements in honey samples.[c]

	Al	B	Ba	Ca	Co	Cr	Cu	Fe	K	Mg	Mn	Mo	Na	Ni	P	Pb	Sn	Sr	Zn
Al	-																		
B	-0.031	-																	
Ba	-0.075	-0.230***	-																
Ca	0.033	0.224**	0.572***	-															
Co	0.195**	0.077	0.367***	0.378***	-														
Cr	0.125	0.042	0.087	0.162*	-0.114	-													
Cu	0.194**	0.274***	0.045	0.234***	0.138*	0.075	-												
Fe	0.307***	0.093	-0.124	-0.044	-0.036	0.282***	0.222**	-											
K	0.067	0.270***	0.366***	0.735***	0.497***	0.083	0.485***	-0.076	-										
Mg	0.090	0.261***	0.404***	0.787***	0.466***	0.152*	0.363***	0.014	0.762***	-									
Mn	0.076	-0.379***	0.745***	0.391***	0.429***	-0.058	-0.020	-0.165*	0.204**	0.376***	-								
Mo	0.107	0.146*	0.236***	0.360***	0.324***	0.230***	0.237***	0.160*	0.460***	0.403***	0.160*	-							
Na	-0.047	0.162*	0.068	0.498***	-0.108	0.229***	0.196**	0.050	0.410***	0.433***	0.054	0.206**	-						
Ni	0.063	0.301***	0.030	0.273***	0.198**	0.262***	0.360***	0.363***	0.310***	0.352***	-0.030	0.248***	0.173*	-					
P	0.121	0.665***	0.076	0.413***	0.253***	0.120	0.465***	0.182**	0.458***	0.483***	-0.127	0.166*	0.156*	0.393***	-				
Pb	0.059	0.079	0.027	0.032	-0.039	0.288***	-0.010	0.478***	-0.075	-0.032	-0.102	0.102	0.049	0.252***	0.022	-			
Sn	-0.120	0.091	-0.001	0.040	-0.089	0.178**	0.135*	0.296***	0.025	-0.026	-0.099	0.193**	-0.015	0.205**	-0.084	0.358***	-		
Sr	0.020	-0.034	0.652***	0.656***	0.369***	0.075	-0.011	-0.041	0.322***	0.546***	0.596***	0.219**	0.248***	0.127	0.131	0.025	-0.017	-	
Zn	-0.121	0.145*	0.172*	0.152*	0.103	0.199**	0.131	0.501***	0.103	0.124	0.002	0.214**	-0.008	0.353***	0.204**	0.622***	0.335***	0.186**	-

[c] Elements Ag, As, Cd, Hg, Sb, Se, and V were not included for this analysis due to the high number of values below the LOR.
*P< 0.05; **P< 0.01; ***P≤ 0.001

Supplementary Table S3. Concentration of 26 elements in honey, by country.

Element	Units	Mean ± SD (range)	Country	Reference
Ag	µg/kg	0.01 - 2.05	Croatia	[1]
		3.82 - 10.25[d]	Thailand	[2]
	mg/kg	0.02 ± 0.04[e]	Greece	[3]
Al	mg/kg	1.55 ± 0.86 (1.22 - 1.76)	Croatia	[4]
		1.6 ± 0.89[e]	Greece	[3]
		1.01 ± 0.76[f]	Greece	[3]
		6.6 (0.21 - 21.3)[g]	New Zealand	[5]
		1.46 - 7.10	Thailand	[2]
		15.3 ± 6.75 (0.775 - 155.6)	Turkey	[6]
	µg/kg	2120 ± 5280 (<10 - 21000)[h]	Australia	[7]
		1400 ± 2300 (264 - 20090)	Italy	[8]
As	µg/kg	13.44 ± 1.33	China	[9]
		1.27 ± 2.88 (0.10 - 18.8)	Italy	[8]
		81.4 ± 117.9 (<0.2 - 533.7)	Saudi Arabia	[10]
		0.54 - 4.19[d]	Thailand	[2]
	mg/kg	0.69 ± 0.21[e]	Greece	[3]
		0.62 ± 0.13[f]	Greece	[3]
		0.068 ± 0.025 (0.027 - 0.126)	Malaysia	[11]
		0.08 (0.04 - 0.17)[g]	New Zealand	[5]
B	mg/kg	5.20 ± 1.75 (2.5 - 10.0)[h]	Australia	[7]
		4.12 ± 2.22[e]	Greece	[3]
		4.01 ± 1.89[f]	Greece	[3]
		6.3 (1.7 - 12.6)	Israel	[12]
		6.7 ± 2.6 (3.6 - 17)	Italy	[13]
		4.42 (0.05 - 0.49)[g]	New Zealand	[5]
Ba	µg/kg	257 ± 145 (85 - 620)[h]	Australia	[7]
		17.6 - 657	Croatia	[1]
		129 ± 151 (6.9 - 945)	Italy	[8]
		147.89 - 1991[d]	Thailand	[2]
	mg/kg	0.22 ± 0.29[e]	Greece	[3]
		0.004 ± 0.01[f]	Greece	[3]
Ca	mg/kg	9.38 ± 2.77 (3.60 - 15.0)[h]	Australia	[7]
		111.24 ± 67.68 (74.37 - 184.39)	Croatia	[4]
		56.97 ± 32.78[e]	Greece	[3]
		54.56 ± 20.01[f]	Greece	[3]
		47.9 ± 40.0 (3.65 - 181)	Hungary	[14]
		84.5 (58.4 - 137.1)	Israel	[12]
		68 ± 42.1 (6.64 - 171.5)	Italy	[8]
		183.67 ± 135.98 (65.80 - 567.27)	Malaysia	[11]
		50.92 (7.21 - 94.3)[g]	New Zealand	[5]
Cd	µg/kg	4.2 ± 0.33	China	[9]
		0.12 - 16.4	Croatia	[1]
		0.61 ± 0.66 (0.05 - 3.21)	Italy	[8]
		11.5 ± 9.4 (<0.3 - 28.0)	Saudi Arabia	[10]
		24.6 - 27.23[d]	Thailand	[2]
	mg/kg	0.35 ± 0.39 (n.d. – 1.03)	Malaysia	[11]

Element	Units	Mean ± SD (range)	Country	Reference
		0.149 (0.01 - 0.45)[g]	New Zealand	[5]
		0.038 ± 0.27 (0.00 - 0.297)	Turkey	[6]
Co	µg/kg	0.74 - 352	Croatia	[1]
		3.96 ± 2.92 (0.74 - 15.1)	Italy	[8]
		0.043 ± 0.028 (n.d. – 0.107)	Malaysia	[11]
		3.0 ± 6.8 (<0.6 - 27.5)	Saudi Arabia	[10]
		0.00 - 96.47[d]	Thailand	[2]
Cr	µg/kg	2.69 - 66.3	Croatia	[1]
		13.3 ± 6.9 (4.80 - 36.7)	Hungary	[14]
		12.8 ± 10.7 (1.58 - 93)	Italy	[8]
		49 ± 51 (10 - 328)	Italy	[13]
		42.9 ± 31.8 (<2.5 - 102.3)	Saudi Arabia	[10]
		102.45 - 132.42[d]	Thailand	[2]
	mg/kg	0.37 (0.12 - 0.55)[g]	New Zealand	[5]
		1.36 ± 1.27 (0.126 - 7.96)	Turkey	[6]
Cu	mg/kg	0.33 ± 0.22 (0.20 - 0.51)	Croatia	[4]
		0.07 - 2.14	Croatia	[1]
		0.82 ± 0.36[e]	Greece	[3]
		0.23 ± 0.19[f]	Greece	[3]
		0.189 ± 0.168 (<0.002 - 0.783)	Hungary	[14]
		0.66 (0.009 - 3.18)	Israel	[12]
		1.96 ± 094 (n.d. – 2.93)	Malaysia	[11]
		0.25 (0.09 - 0.70)[g]	New Zealand	[5]
		0.14 - 4.77[d]	Thailand	[2]
		11.03 ± 10.03 (0.223 - 198.36)	Turkey	[6]
	µg/kg	203 ± 168 (65 - 770)[h]	Australia	[7]
		46.18 ± 3.91	China	[9]
		220 ± 134 (69 - 763)	Italy	[8]
		15.7 ± 52.2 (<0.1 - 238.4)	Saudi Arabia	[10]
Fe	mg/kg	1.44 ± 0.94 (0.58 - 3.70)[h]	Australia	[7]
		1.23 ± 0.95 (0.92 - 1.38)	Croatia	[4]
		2.94 ± 1.51[e]	Greece	[3]
		1.39 ± 0.55[f]	Greece	[3]
		0.76 ± 0.758 (<0.005 - 2.86)	Hungary	[14]
		2.9 (0.9 - 9.3)	Israel	[12]
		2.08 ± 1.06 (0.624 - 6.19)	Italy	[8]
		162.31 ± 49.21 (55.83 - 233.0)	Malaysia	[11]
		1.71 (0.67 - 3.39)[g]	New Zealand	[5]
		107.8 ± 20.0 (3.51 - 1278.8)	Turkey	[6]
Hg	µg/kg	1.65 ± 0.14	China	[9]
		0.19 ± 0.27 (0.04 - 1.46)	Italy	[8]
K	mg/kg	8370 ± 3560 (2500 - 15800)[h]	Australia	[7]
		325.54 ± 108.55 (258.69 - 360.80)	Croatia	[4]
		372 ± 270 (62.4 - 1158)	Hungary	[14]
		1153.7 (189.0 - 3768.3)	Israel	[12]
		1450 ± 1100 (237 - 6520)	Italy	[13]
		1349.34 ± 971.53 (413.63 - 4026.40)	Malaysia	[11]
		1050 (34.8 - 3640)[g]	New Zealand	[5]

Element	Units	Mean ± SD (range)	Country	Reference
Mg	mg/kg	40.1 ± 18.32 (16.0 - 87.0)[h]	Australia	[7]
		22.01 ± 10.62 (16.82 - 30.68)	Croatia	[4]
		62.88 ± 2.99[e]	Greece	[3]
		15.88 ± 9.23[f]	Greece	[3]
		16.3 ± 9.67 (<0.104 - 35.1)	Hungary	[14]
		64.0 (18.5 - 204.6)	Israel	[12]
		34 ± 25 (6.2 - 148)	Italy	[13]
		64.46 ± 57.74 (21.83 - 199.33)	Malaysia	[11]
		24.8 (7.52 - 86.3)	New Zealand	[5]
		7.90 - 32.99[d]	Thailand	[2]
		50.5 ± 7.6 (5.83 - 309.8)	Turkey	[6]
Mn	mg/kg	4.23 ± 2.29 (0.89 - 10.0)[h]	Australia	[7]
		0.17 ± 0.10 (0.15 - 0.19)	Croatia	[4]
		2.76 ± 4.54[e]	Greece	[3]
		0.17 ± 0.08[f]	Greece	[3]
		1.03 ± 0.89 (0.026 - 4.23)	Hungary	[14]
		0.76 ± 0.68 (0.09 - 2.8)	Italy	[13]
		0.664 ± 0.829 (0.049 - 4.375)	Italy	[8]
		1.04 (0.18 - 4.75)[g]	New Zealand	[5]
		0.15 - 2.68[d]	Thailand	[2]
		1.99 ± 3.6 (0.096 - 29.5)	Turkey	[6]
Mo	µg/kg	0.54 - 348	Croatia	[1]
		30 ± 70[e]	Greece	[3]
		27.8 ± 17.8 (2.15 - 66.2)	Hungary	[14]
		7.14 ± 13.5 (1.93 - 81)	Italy	[8]
Na	mg/kg	137 ± 122 (21.0 - 510)[h]	Australia	[7]
		95.85 ± 53.00 (51.62 - 168.86)	Croatia	[4]
		13.0 - 156	Croatia	[1]
		61.6 (26.6 - 132.5)	Israel	[12]
		45 ± 46 (4.8 - 176)	Italy	[13]
		236.8 ± 177.57 (83.17 - 732.16)	Malaysia	[11]
		23.9 (1.10 - 110)[g]	New Zealand	[5]
Ni	µg/kg	33 ± 42 (<10 - 170)[h]	Australia	[7]
		46.9 ± 39.2 (<2.8 - 191.0)	Saudi Arabia	[10]
		12.54 - 1455.09[d]	Thailand	[2]
	mg/kg	0.45 ± 0.30 (0.16 - 0.98)	Croatia	[4]
		0.41 ± 0.40[e]	Greece	[3]
		0.03 ± 0.03[f]	Greece	[3]
		0.14 ± 0.09 (0.05 - 0.40)	Italy	[13]
		0.23 (0.02 - 0.65)[g]	New Zealand	[5]
P	mg/kg	46.4 ± 15.7 (31.0 - 98.0)[h]	Australia	[7]
		44.3 ± 22.9 (19.7 - 93.5)	Hungary	[14]
		184.7 (47.0 - 651)	Israel	[12]
		75 ± 37 (17 - 207)	Italy	[13]
		46.0 (29.5 - 255)[g]	New Zealand	[5]
Pb	µg/kg	33.98 ± 2.88	China	[9]
		32.8 ± 47.9 (0.85 - 370)	Italy	[8]
		73.0 ± 72.3 (<1.7 - 240.0)	Saudi Arabia	[10]

Element	Units	Mean ± SD (range)	Country	Reference
		82.52 - 1066.17[d]	Thailand	[2]
	mg/kg	0.05 ± 0.04 (0.04 - 0.08)	Croatia	[4]
		0.18 ± 0.14[e]	Greece	[3]
		0.16 ± 0.13[f]	Greece	[3]
		2.56 (0.15 - 8.22)	Israel	[12]
		0.36 ± 0.39 (n.d. – 1.017)	Malaysia	[11]
		0.017 (0.01 - 0.04)[g]	New Zealand	[5]
		0.349 ± 0.81 (0.00 - 3.04)	Turkey	[6]
Sb	µg/kg	0.23 - 4.02	Croatia	[1]
		0.42 ± 0.69 (0.13 - 5.84)	Italy	[8]
	mg/kg	0.49 ± 0.15[e]	Greece	[3]
		0.60 ± 0.16[f]	Greece	[3]
Se	µg/kg	0.16 - 36.3	Croatia	[1]
		13.2 ± 7.38 (2.66 - 36.4)	Hungary	[14]
		1.46 ± 1.62 (0.64 - 7.88)	Italy	[8]
		47 ± 11 (33 - 74)	Italy	[13]
		12.33 - 98.75[d]	Thailand	[2]
	mg/kg	0.42 ± 0.26[e]	Greece	[3]
		0.16 ± 0.20[f]	Greece	[3]
		2.18 ± 1.67 (0.418 - 19.9)	Turkey	[6]
Sn	µg/kg	21 ± 14 (<10 - 55)[h]	Australia	[7]
		25.2 ± 30.1 (4.4 - 222)	Italy	[8]
		35 ± 59 (10 - 246)	Italy	[13]
Sr	µg/kg	683 ± 564 (150 - 3100)[h]	Australia	[7]
		232 ± 217 (24.8 - 1557)	Italy	[8]
		1.17 - 476.75[d]	Thailand	[2]
V	µg/kg	0.96 - 75.8	Croatia	[1]
		2.34 ± 2.21 (0.14 - 10.4)	Italy	[8]
		5.86 - 12.71[d]	Thailand	[2]
Zn	mg/kg	1.46 ± 3.75 (0.33 - 19.0)[h]	Australia	[7]
		7.12 ± 4.95 (1.61 - 16.46)	Croatia	[4]
		1.24 ± 1.25[e]	Greece	[3]
		1.00 ± 0.46[f]	Greece	[3]
		2.32 ± 1.84 (0.185 - 7.2)	Hungary	[14]
		4.1 (0.8 - 11.5)	Israel	[12]
		43.88 ± 46.58 (4.70 - 173.77)	Malaysia	[11]
		1.18 (0.20 - 2.46)[g]	New Zealand	[5]
		18.2 ± 9.12 (1.734 - 245.2)	Turkey	[6]
	µg/kg	1329.5 ± 112.2	China	[9]
		1072 ± 1315 (285 - 11230)	Italy	[8]
		1708 ± 747 (598 - 4190)	Saudi Arabia	[10]

[d] Range values of means;

[e] Pine honeys only;

[f] Thyme honeys only;

[g] Values of means and ranges;

[h] Australian mainland honeys only;

References

1. Bilandzic, N.; Tlak Gajger, I.; Kosanovic, M.; Calopek, B.; Sedak, M.; Solomun Kolanovic, B.; Varenina, I.; Luburic, D.B.; Varga, I.; Dokic, M. Essential and toxic element concentrations in monofloral honeys from southern Croatia. *Food Chem.* **2017**, *234*, 245-253, doi:10.1016/j.foodchem.2017.04.180.
2. Wetwitayaklung, P.; Wangwattana, B.; Narakornwit, W. Determination of trace-elements and toxic heavy minerals in Thai longan, litchi and Siam weed honeys using ICP-MS. *Int. Food Res. J.* **2018**, *25*, 1464-1473.
3. Karabagias, I.K.; Louppis, A.P.; Kontakos, S.; Papastephanou, C.; Kontominas, M.G. Characterization and geographical discrimination of Greek pine and thyme honeys based on their mineral content, using chemometrics. *Eur. Food Res. Technol.* **2017**, *243*, 101-113, doi:10.1007/s00217-016-2727-8.
4. Uršulin-Trstenjak, N.; Puntarić, D.; Levanić, D.; Gvozdić, V.; Pavlek, Ž.; Puntarić, A.; Puntarić, E.; Puntarić, I.; Vidosavljević, D.; Lasić, D., et al. Pollen, Physicochemical, and Mineral Analysis of Croatian Acacia Honey Samples: Applicability for Identification of Botanical and Geographical Origin. *J. Food Qual.* **2017**, *2017*, 1-11, doi:10.1155/2017/8538693.
5. Vanhanen, L.P.; Emmertz, A.; Savage, G.P. Mineral analysis of mono-floral New Zealand honey. *Food Chem.* **2011**, *128*, 236-240, doi:10.1016/j.foodchem.2011.02.064.
6. Altunatmaz, S.S.; Tarhan, D.; Aksu, F.; Ozsobaci, N.P.; Or, M.E.; BarutÇU, U.B. Levels of Chromium, Copper, Iron, Magnesium, Manganese, Selenium, Zinc, Cadmium, Lead and Aluminium of honey varieties produced in Turkey. *Food Sci. Technol. (Campinas)* **2019**, *39*, 392-397, doi:10.1590/fst.19718.
7. Zhou, X.; Taylor, M.P.; Salouros, H.; Prasad, S. Authenticity and geographic origin of global honeys determined using carbon isotope ratios and trace elements. *Sci. Rep.* **2018**, *8*, 14639, doi:10.1038/s41598-018-32764-w.
8. Quinto, M.; Miedico, O.; Spadaccino, G.; Paglia, G.; Mangiacotti, M.; Li, D.; Centonze, D.; Chiaravalle, A.E. Characterization, chemometric evaluation, and human health-related aspects of essential and toxic elements in Italian honey samples by inductively coupled plasma mass spectrometry. *Environ. Sci. Pollut. Res. Int.* **2016**, *23*, 25374-25384, doi:10.1007/s11356-016-7662-5.
9. Ru, Q.M.; Feng, Q.; He, J.Z. Risk assessment of heavy metals in honey consumed in Zhejiang province, southeastern China. *Food Chem. Toxicol.* **2013**, *53*, 256-262, doi:10.1016/j.fct.2012.12.015.
10. Bazeyad, A.Y.; Al-Sarar, A.S.; Rushdi, A.I.; Hassanin, A.S.; Abobakr, Y. Levels of heavy metals in a multifloral Saudi honey. *Environ. Sci. Pollut. Res. Int.* **2019**, *26*, 3946-3953, doi:10.1007/s11356-018-3909-7.
11. Moniruzzaman, M.; Chowdhury, M.A.; Rahman, M.A.; Sulaiman, S.A.; Gan, S.H. Determination of mineral, trace element, and pesticide levels in honey samples originating from different regions of Malaysia compared to manuka honey. *Biomed. Res. Int.* **2014**, *2014*, 359890, doi:10.1155/2014/359890.
12. Dag, A.; Afik, O.; Yeselson, Y.; Schaffer, A.; Shafir, S. Physical, chemical and palynological characterization of avocado (Persea americana Mill.) honey in Israel. *Int. J. Food Sci. Technol.* **2006**, *41*, 387-394, doi:10.1111/j.1365-2621.2005.01081.x.
13. Conti, M.E.; Canepari, S.; Finoia, M.G.; Mele, G.; Astolfi, M.L. Characterization of Italian multifloral honeys on the basis of their mineral content and some typical quality parameters. *J. Food Compos. Anal.* **2018**, *74*, 102-113, doi:10.1016/j.jfca.2018.09.002.
14. Czipa, N.; Andrasi, D.; Kovacs, B. Determination of essential and toxic elements in Hungarian honeys. *Food Chem.* **2015**, *175*, 536-542, doi:10.1016/j.foodchem.2014.12.018.
